# Sarcopenia is associated with early postoperative cognitive decline in older adults following hip fracture surgery: a prospective cohort study

**DOI:** 10.1186/s12877-025-06811-x

**Published:** 2025-12-02

**Authors:** Tao Jiang, Pingjuan Wang, Jianxiao Wu, Xiangnan Liang, Yiqiao Wang, Xianwen Hu

**Affiliations:** 1 Department of Anesthesiology, The Second Affiliated Hospital of Anhui Medical University, No.678 Furong Road, Hefei, Anhui Province 230601 China; 2 Department of Anesthesiology, Anhui No.2 Provincial People’s Hospital, Hefei, Anhui Province 230041, China; 3 Key Laboratory of Anesthesiology and Perioperative Medicine of Anhui Higher Education Institutes, Anhui Medical University, Hefei, Anhui Province 230601 China

**Keywords:** Aged, Risk factors, Sarcopenia, Postoperative cognitive, Hip fracture surgery

## Abstract

**Background:**

Early postoperative cognitive decline (POCD) frequently affects elderly individuals with hip fractures, and a significant number of these patients exhibit sarcopenia prior to surgery. Identifying patients with characteristics associated with an increased risk of early POCD at an early stage is essential for managing it effectively. Sarcopenia is a condition linked to aging, marked by a progressive reduction in muscle mass and function. Previous studies revealed that having lower muscle mass is linked to the development of perioperative neurocognitive disorders. This research aims to discover the association between sarcopenia and early POCD in aged hip fractures patients.

**Methods:**

The study is a single-center, observational cohort study of patients aged 65 and above who underwent hip fracture surgery and were enrolled. Based on the Asian Working Group for Sarcopenia (AWGS) 2019 criteria, sarcopenia was diagnosed when both handgrip strength and skeletal muscle mass (measured by the axial plane of chest CT at the level of the T12)were below the established diagnostic cutoffs. Early POCD was characterized by a decrease of at least 1.5 standard deviations (SDs) in the results of two or more neuropsychological tests on postoperative day 7. The relationship between sarcopenia and early cognitive decline after surgery was examined using multivariate logistic regression, while its discriminatory ability was evaluated through the area under the receiver operating characteristic curve (AUC_ROC_).

**Results:**

A total of 132 patients were included, 35.6% of whom had preoperative sarcopenia. Early POCD was observed in thirty patients (22.7%), as indicated by a reduction in their scores on the Trail Making Tests, Digit Symbol Substitution Test, and Clock Drawing Test. Multivariable analysis showed that preoperative sarcopenia(OR = 3.716, 95% CI:1.618–8.814, *P* = 0.005) was independently linked to early POCD after adjusting for age 80 and above (OR = 3.364, 95% CI:1.343–8.427, *P* = 0.01), and high-sensitivity C-reactive protein (Hs-CRP) > 6 mg/L(OR = 3.229, 95% CI:1.308–7.968, *P* = 0.011). The AUC_ROC_ was 0.658 (95% CI: 0.543–0.772). Furthermore, when combining the age ≥ 80 years in the model, the AUC_ROC_ reached 0.704(95% CI: 0.584–0.823, *P* = 0.001), indicating enhanced discriminatory performance in identifying patients at risk.

**Conclusion:**

Sarcopenia, as defined by AWGS2019 criteria was independently associated with early POCD in elderly individuals after hip fracture surgery. When combined with age ≥ 80 years, it improves the identification of patients at high risk for early POCD.

**Trial registration:**

ChiCTR2200055540, January 11, 2022.

## Background

Sarcopenia is a state related to frailty which mainly has an impact on elderly people, and is marked by a decrease in skeletal muscle mass, strength, and physical activity levels [1]. Although frailty and sarcopenia share broad clinical similarities, sarcopenia was recognized as an independent condition through the International Classification of Diseases-10 code in 2016 [2, 3]. Elderly individuals with sarcopenia are more susceptible to falls, which can result in hip fractures [4]. Hip fractures are a source of concern for people older than 60 years, as they are linked to increased risks of premature death, considerable disability, and a decline in functional status [5]. Especially, sarcopenia is linked to more severe cognitive impairment in this age group [6]. Surgical intervention plays a crucial role in managing hip fractures among elderly patients. However, the trauma associated with surgery can substantially elevate the likelihood of postoperative cognitive impairment [7]. This condition is defined by deficits in various cognitive abilities, such as awareness, memory, attention, psychomotor skills, and learning capacity, which are distinct from the cognitive dysfunctions associated with delirium and dementia [8]. The frequency of cognitive decline ranges from 6.7% to 75% in hip fracture patients one week after surgery [9]. The pathogenesis of early postoperative cognitive decline (POCD) is multifactorial, with mutually interdependent mechanisms that include surgery-, anesthesia-, and patient-related risk factors underlying its development.

Aging is the main pathogenic factor for sarcopenia and early POCD, involving mechanisms such as inflammation and dysfunction of lipid metabolism [10]. Recent research has shown that administering corticosteroids before surgery can lessen the inflammatory reaction caused by surgical procedures. This, in turn, leads to a reduction in both the frequency and severity of early POCD [11]. Moreover, chronic inflammation assumes a vital role in the initiation and advancement of sarcopenia [12]. Sarcopenia facilitates brain inflammatory responses, influences the progression of cognitive decline via the muscle-brain axis and reduces mitochondrial function [13]. Our study integrates high-sensitivity C-reactive protein (Hs-CRP) measurements with sarcopenia assessment, offering a more comprehensive evaluation of the inflammatory and metabolic factors associated with POCD. Ceramides, which are bioactive sphingolipids, accumulate extensively in aged muscles [14]. However, by measuring the plasma ceramide concentrations, some researchers have found that increased concentrations predict memory loss, reduced hippocampal morphology and the occurrence of neuroinflammation [15]. Whether increased ceramide concentration in elderly patients with sarcopenia is a risk factor for early POCD is the main content of this study. At the same time, inflammation may affect both muscle-brain signaling pathway, which constitutes the potential link between sarcopenia and cognitive dysfunction. This association continues to be a key focus of the study.

An earlier animal study suggested that the extent of muscle mass reduction was linked to a higher occurrence of perioperative neurocognitive impairment in rats [16]. Furthermore, numerous studies have indicated that a low level of handgrip strength (HSG) is linked to an elevated risk of postoperative delirium [17]. According to the Asian Working Group for Sarcopenia (AWGS) 2019 report, diagnosing sarcopenia requires evidence of both diminished HSG and reduced skeletal muscle mass. We utilized the AWGS 2019 criteria combined with CT-based T12 measurements to assess sarcopenia, providing a more accurate and up-to-date diagnostic approach. Nonetheless, as far as we are aware, the relationship between sarcopenia, as outlined by AWGS 2019 [18], and early POCD has yet to be explored. Therefore, our aim was to evaluate the relationship between sarcopenia and early POCD, specifically examining the role of sarcopenia in relation to early cognitive decline following hip fracture surgery in elderly patients.

## Methods

### Ethical approval

A total of 132 patients were enlisted subsequent to registration on www.chictr.org.cn (ChiCTR2200055540, date of registration: January 11, 2022, principal investigator: Tao Jiang), in accordance with the protocol. This observational cohort study was approved by the ethical committee of Anhui No.2 Provincial People’s Hospital, China((R)2021-004). All patients provided written informed consent, and the perioperative neurocognitive evaluations were conducted by the same team composed of residents and attending physicians.

### Inclusion and exclusion criteria

From January 2022 to January 2023, elderly patients undergoing hip fracture (defined as fracture of the proximal femur) surgery at Anhui No.2 Provincial People’s Hospital were eligible to participate. All patients underwent preoperative examinations, chest CT scans and surgical treatments within 3 days of admission. Inclusion criteria included: (1) age ≥ 65 years of age or older; (2) an isolated hip fracture resulting from a fall; (3) American Society of Anesthesiologists (ASA) II or III; (4) interaction and completion of neuropsychological tests. The exclusion criteria were: (1) baseline Mini-Mental State Examination (MMSE) score of 23 or lower; (2) pathological fractures or multiple fractures, (3) failure to completing the sarcopenia or neuropsychological tests assessment.

### Data collection

All patients’ basic data were collected by a resident doctor according to the fixed template content in the research report. The neurocognitive function and anxiety/depression of all patients underwent screening. We also collected data from the pre-operation laboratory, including high-sensitivity C-reactive protein (Hs-CRP) levels, plasma ceramide (16:0), plasma ceramide (22:0) and plasma ceramide (24:0) levels. The intraoperative data included the durations of surgery and anesthesia, anesthetic maintenance drugs, transfusion volume, and the use of patient-controlled intravenous analgesia.

### Measurement of sarcopenia

Sarcopenia was identified using the guidelines set forth by AWGS2019. A diagnosis of sarcopenia necessitated both diminished HSG and reduced skeletal muscle mass, as determined by the skeletal muscle mass index at the 12th thoracic vertebra. Muscle strength was evaluated by determining the maximum grip force of the patient through the use of a dynamometer (CAMRY, EH101) when held with one hand. Patients were in the supine position and encouraged to exert the greatest force possible; they were asked to apply maximum HSG on two occasions, using both their left and right hand, and the dominant hand was measured. This study defined low muscle strength as HGS < 28 kg for men and < 18 kg for women [19]. The next step is to calculate the skeletal muscle index (SMI). CT images were uploaded into PACS (Neusoft Corp., China) [20]. The total skeletal muscle area (SMA) was assessed in the axial plane of chest CT at the level of the T12 vertebra [21]. The SMA was delineated on a single CT scan using the established Hounsfield Unit (HU) range of − 29 to 150, encompassing key muscle groups such as the erector spinae, latissimus dorsi, rectus abdominis, external and internal oblique muscles, and both the internal and external intercostal muscles. The SMI was calculated as SMI = SMA/height(m)^2. Muscle mass deficiency was identified when the SMI was ≤ 42.6 cm²/m² for men and ≤ 30.6 cm²/m² for women, based on reference [22].

Based on AWGS2019 criteria, the presence of sarcopenia was defined as the combination of diminished HSG (< 28 kg for men and < 18 kg for women) and reduced SMI (≤ 42.6 cm²/m² for men and 30.6 cm²/m² for women). A total of 132 patients demonstrated reduced HSG in this study. Among them, 47 patients with reduced SMI were classified into the sarcopenia group, whereas the remaining 85 patients were assigned to the non-sarcopenia group.

All CT scans were evaluated by a single experienced researcher who was unaware of the outcomes. The evaluation of muscle mass is conducted through CT imaging of the SMI at the 12th thoracic vertebra. Furthermore, in the year of the study project, CT scans are extensively utilized in clinical lung diseases screening for ‌Corona Virus Disease 2019 and do not increase overall costs.

### Surgery and anesthesia

Before the surgical procedure, patients were advised to abstain from food for 8 h and clear liquids for 4 h, with no preoperative medications administered. Upon arrival in the operating theatre, standard monitoring protocols were initiated, including electrocardiography, invasive arterial blood pressure monitoring, and pulse oximetry. Anesthesia induction was achieved through intravenous administration of midazolam at 0.02 mg/kg, sufentanil at 0.5 µg/kg, etomidate at 0.3 mg/kg, and rocuronium at 0.6 mg/kg. Once patient stability was confirmed, tracheal intubation was performed and connected to an anesthesia machine configured with the following settings: tidal volume ranging from 6 to 8 ml/kg, fractional inspired oxygen set at 80%, an inspiratory-to-expiratory ratio of 1:2, and a respiratory rate of 12 breaths per minute.

During the maintenance phase of anesthesia, propofol was continuously infused intravenously at a rate of 4–6 mg/kg/h, while remifentanil was delivered at 0.1–0.2 µg/kg/min. The propofol infusion rate was adjusted to maintain a bispectral index between 40 and 50. Muscle relaxation was maintained by administering intermittent intravenous boluses of cisatracurium at 0.03 mg/kg once the post-tetanic count reached or exceeded 3 following strong stimulation by the muscle relaxant monitor.

Throughout the surgery, blood transfusions were administered if hemoglobin levels dropped to or below 70 g/L. In cases where heart rate decreased to or below 45 beats per minute, 0.5 mg of atropine was given intravenously. If systolic blood pressure fell by more than 30% from baseline or dropped to 90 mmHg or lower, 6 mg of ephedrine was administered intravenously.

### Diagnosis of early POCD

Eligible participants completed the Self-rating Depression (SDS) test, the Self-rating Anxiety Scale (SAS) test, and MMSE, and those with severe depression or cognitive impairment were excluded. There is no internationally accepted evaluation for early POCD. Thus, various neuropsychological functions (e.g. attention, concentration, and visuospatial ability) have been evaluated at the bedside to screen for neurocognitive disorders, such as delirium [23]. The participants enrolled in the study completed a range of neuropsychological assessments administered by the same team before the operation and again on the seventh day after the operation. Although assessors were not formally blinded to the participants’ clinical status, the cognitive assessments were conducted using objective and standardized tests with predefined scoring criteria to minimize subjective interpretation and potential bias. To ensure high inter-assessor reliability, all evaluators completed mandatory standardized training and practiced on non-study volunteers until a consistent scoring consensus was achieved across all assessments. While no consensus has been established regarding the timing of cognitive assessments after surgery, several studies have suggested an optimal timing at 1week and 3 months post-procedure [24–28]. Postoperative day 7 was chosen for its alignment with previous studies [27, 29], aiming to capture early cognitive decline while minimizing loss to follow-up.

Three areas of cognitive function are evaluated by means of the neuropsychological tests: attention, executive function and memory. The selected tests were selected to cover key cognitive domains known to be vulnerable in the postoperative period:

- Attention and working memory: assessed using the Digit Span Test (DST).
- Processing speed, visual-motor coordination, and executive function: assessed using the Digit Symbol Substitution Test (DSST) and the Trail Making Tests (TMTs).
- Visuospatial construction and executive function: measured using the Clock Drawing Test (CDT).

On the seventh day after the surgery, individuals who got high scores are more likely to have good cognitive performance, like in tests such as DST, DSST and CDT. The TMTs assessed attentional and executive functioning, with shorter times reflecting greater processing speed, visual-motor coordination, and executive function.

In this study, we adopted a definition based on a decline in performance from a patient’s own preoperative baseline on a battery of neuropsychological tests, as has been used in previous investigations of postoperative cognitive changes [27, 30]. Neuropsychological testing is designed to assess whether a one standard deviation decrease from preoperative testing indicates postoperative neuropsychological impairment. The standard deviation for each test was derived from the pre-surgery evaluations of all the patients. For each participant, we calculated the difference between the postoperative and preoperative scores, then divided this difference by the respective standard deviation to derive a Z score for every individual test. These Z scores were subsequently utilized to evaluate early POCD. Patients were identified as having postoperative cognitive impairment if their Z score fell below 1.5 standard deviations on at least two out of the four neuropsychological assessments [31–34].

### Sample size calculation

This study employed a logistic regression models events per variable (EPV) for estimating the minimum sample size, where each variable demanded a minimum of 10 events. Based on prior literature [35, 36], we identified three key variables anticipated to be included in the final multivariate model: increasing age, Hs-CRP, and sarcopenia. The formula for estimating the required sample size using the EPV approach is as follows: Sample size = (Number of variables × EVP)/Event occurrence rate, where EVP is typically set to 10 [37]. The reported occurrence rate of cognitive dysfunction subsequent to hip surgery was 25.8% [38], considering a 10% dropout rate, at least 130 patients were necessary to have three variables in the final multi-variable model.

### Statistical analysis

The statistical characteristics of the variables were analyzed for both sarcopenia and non-sarcopenia patients. Numerical data are reported as means ± standard deviation, while categorical data are shown as frequency (percentage). The statistical disparities between the sarcopenia and non-sarcopenia groups were assessed using t-tests for normally distributed data, while the Mann-Whitney U test was applied for data that did not follow a normal distribution. The qualitative data were evaluated using the chi-square test or Fisher’s exact test. The relationship between each variable and early POCD was examined through univariate regression analysis. Moreover, factors with a *P*-value less than 0.05 in the single-variable analysis were incorporated into the multiple-variable analysis (Enter) to determine the independent predictors of cognitive decline after surgery. Following this, a multicollinearity assessment was conducted by calculating the tolerance levels and variance inflation factor (VIF). Tolerance levels exceeding 0.1 and VIF values below 10 were interpreted as indicating an absence of multicollinearity among the independent variables. The findings from the multivariate analysis are displayed as odds ratios (ORs) alongside 95% confidence intervals (CIs). Furthermore, a receiver operating characteristic (ROC) curve was employed to evaluate the predictive power of preoperative sarcopenia and sarcopenia + age ≥ 80 years for early POCD. A *P*-value set as two-sided and below 0.05 was considered statistically significant.

### Results

**Fig. 1:**
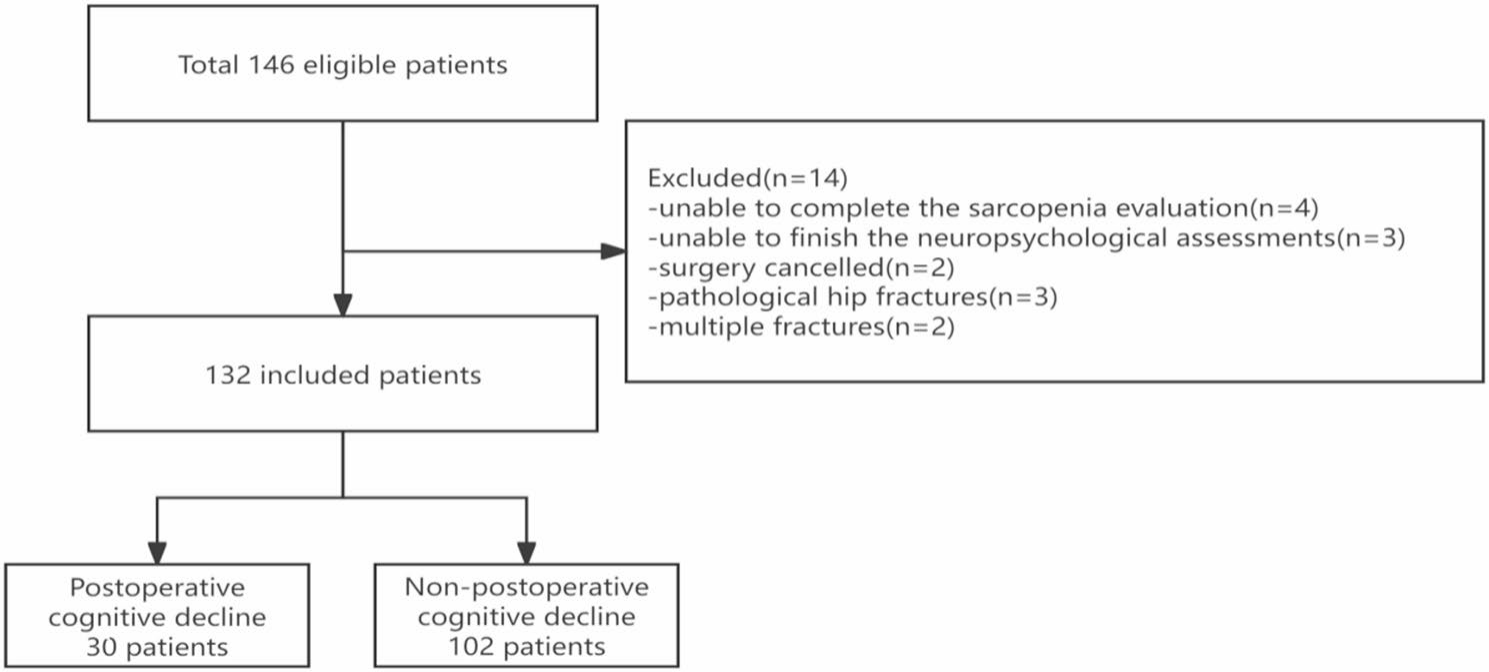
Flow chart of the details of the study. The flow chart of the patients that are included in the study

In this study, a total of 146 patients who underwent hip fracture surgery were considered for inclusion. However, 14 patients were excluded based on the following six criteria: unable to complete the sarcopenia evaluation (*n* = 4), unable to finish the neuropsychological assessments (*n* = 3), surgery cancelled (*n* = 2), pathological hip fractures(*n* = 3), and multiple fractures (*n* = 2). Consequently, the final analysis included the remaining 132 patients (Fig. 1).

### Patient characteristics

Table 1 outlines the attributes of the patients, contrasting those identified with sarcopenia against those without. A total of forty-seven individuals were found to have sarcopenia. In contrast to individuals without sarcopenia, those affected by the condition were typically older (*P* = 0.038), exhibited higher rates of hypertension (*P* = 0.045) and diabetes mellitus (*P* = 0.031), and possessed a lower body mass index (BMI) (*P* = 0.002). In contrast to those without sarcopenia, individuals with the condition displayed multiple irregularities, such as increased Hs-CRP levels (*P* = 0.002), and higher preoperative plasma concentrations of ceramide (22:0) (*P* = 0.025).

**Table 1:** Demographic characteristics of the patients with sarcopenia and without sarcopenia

Demographics	ALL(*n* = 132)	Sarcopenia(*n* = 47)	Non-Sarcopenia(*n* = 85)	*P* value
Age (Year)	77 ± 7.28	78.77 ± 6.9	76.02 ± 7.34	**0.038**
Male sex, *n* (%)	44(33.3)	17(36.2)	27(31.8)	0.607
BMI (kg/m^2^)	22.90 ± 2.61	21.99 ± 2.85	23.41 ± 2.33	**0.002**
Educational level, *n* (%)				0.563
< 5 years	89(67.4)	30(63.8)	59(69.4)	
≥ 5 years	43(32.6)	17(36.2)	26(30.6)	
Smoking history, *n* (%)	35(26.5)	12(25.5)	23(27.1)	0.849
Drinking history, *n* (%)	16(12.1)	5(10.6)	11(12.9)	0.698
Comorbidities, *n* (%)				
Hypertension	76(57.6)	33(70.2)	43(50.6)	**0.045**
Diabetes mellitus	37(28.0)	19(40.4)	18(21.2)	**0.031**
Coronary heart disease	23(17.4)	9(19.1)	14(16.5)	0.698
COPD	16(12.1)	5(10.6)	11(12.9)	0.698
Cerebrovascular disease	17(12.9)	8(17.0)	9(10.6)	0.291
ASA physical status, *n* (%)				0.586
Class II	67(50.8)	22(46.8)	45(52.9)	
Class III	65(49.2)	25(53.2)	40(47.1)	
Preoperative MMSE	26.31 ± 1.62	26.17 ± 1.71	26.39 ± 1.57	0.461
Baseline SAS score	23.1 ± 2.78	22.6 ± 3.16	23.3 ± 2.52	0.230
Baseline SDS score	24.3 ± 2.67	24.0 ± 3.16	24.5 ± 2.36	0.354
Type of surgery, *n* (%)				0.98
Femoral neck facture	52(39.4)	18(38.3)	34(40.0)	
Intertrochanteric fracture	41(31.1)	15(31.9)	26(30.6)	
Subtrochanteric fracture	39(29.5)	14(29.8)	25(29.4)	
Hs-CRP, (mg/L)	6.36 ± 1.67	6.96 ± 1.64	6.03 ± 1.61	**0.002**
Plasm ceramide(nmol/L)				
Plasm ceramide (24:0)	147.88 ± 11.74	149.99 ± 12.51	146.71 ± 11.20	0.138
Plasm ceramide (22:0)	19.28 ± 3.63	20.22 ± 4.01	18.75 ± 3.30	**0.025**
Plasm ceramide (16:0)	29.73 ± 2.83	30.06 ± 2.24	29.55 ± 3.12	0.277

Significant values are in [bold]

*BMI* Body mass index, *COPD* Chronic obstructive pulmonary disease, *ASA* American society of Anaesthesiologists, *MMSE* Mini-Mental State Examination, *SAS* the Self-rating Anxiety Scale, *SDS* the Self-rating Depression Scale, *Hs-CRP* High-sensitivity C-reactive protein

Table 2 show the intraoperative data of the patients with sarcopenia and without sarcopenia. There was no statistically significant difference between the two groups with respect to the duration of operation, anesthesia time, infusion volume, the usage amounts of propofol, remifentanil, and cisatracurium, and the case of uses of atropine and ephedrine (*P* > 0.05).

**Table 2:** The intraoperative data of the patients with sarcopenia and without sarcopenia

Demographics	ALL(*n* = 132)	Sarcopenia(*n* = 47)	Non-Sarcopenia(*n* = 85)	*P* value
Infusion volume(ml)	1302.12 ± 184.02	1310.64 ± 205.61	1297.41 ± 172.04	0.458
Propofol(mg)	502.79 ± 37.93	502.51 ± 40.93	502.94 ± 36.41	0.133
Remifentanil(µg)	1460.67 ± 104.78	1432.77 ± 95.82	1476.09 ± 106.85	0.364
Cisatracurium(mg)	8.39 ± 1.16	8.27 ± 1.20	8.47 ± 1.13	0.591
Atropine cases (%)	49(37.1)	15(31.9)	34(40)	0.357
Ephedrine cases (%)	62(47)	20(42.6)	42(49.4)	0.450
Operation time(min)	125.63 ± 9.78	122.66 ± 9.35	127.67 ± 9.67	0.826
Anesthesia time (min)	173.06 ± 9.99	175.74 ± 10.01	171.58 ± 9.72	0.973

### Neuropsychological test results

The preoperative SAS and SDS scores were identical for both patient groups (Table 1).

On the seventh day following surgery, thirty individuals (22.7%) were identified as experiencing cognitive decline. The occurrence of this condition was statistically more frequent among those with sarcopenia compared to those without (38.3%vs.14.1%, *P* = 0.003). Furthermore, the cognitive performance of the sarcopenia group, as measured by the TMTs, DSST, and CDT, showed a significant decline on the seventh postoperative day compared to their preoperative baseline scores (*P* < 0.05). The neuropsychological assessment outcomes for the participants at the initial stage and on the seventh day following surgery are detailed in.

**Fig. 2:**
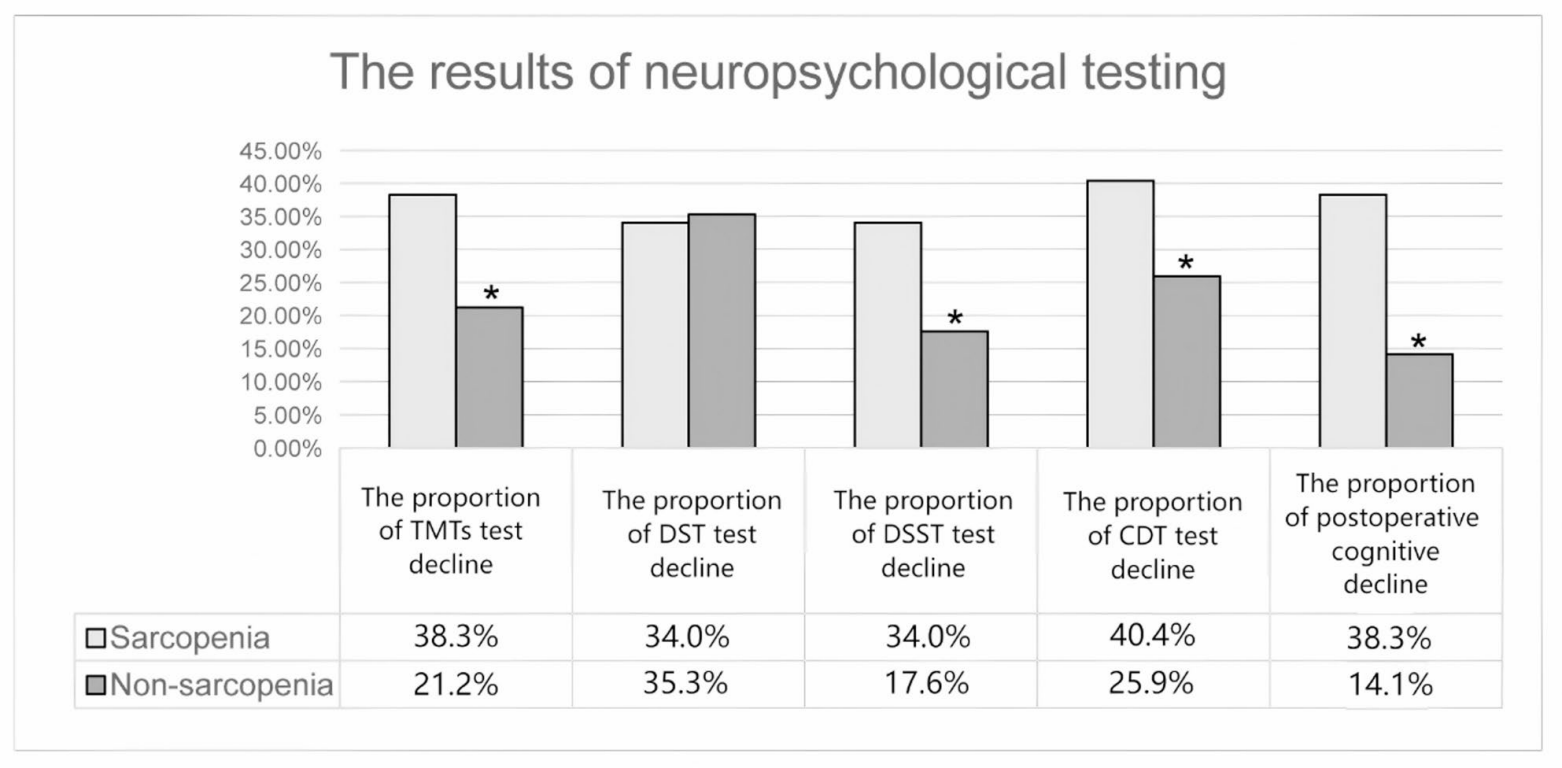
Results of neuropsychological testing. Comparison of four cognitive domains and early POCD between sarcopenia group(*n* = 47) and non-sarcopenia group(*n* = 85) with ^*^P < 0.05. TMTs: the trail making tests, DST: digit span test, DSST: digit symbol substitution, CDT: clock drawing test

Table 3. Those with sarcopenia exhibited lower performance on the TMTs, DSST, and CDT (*P* < 0.05) (Fig. 2).

**Table 3:** Neuropsychological test results at baseline and on postoperative day 7

	Baseline			Postoperative day 7		
	Sarcopenia (*n* = 47)	Non-Sarcopenia (*n* = 85)	*P* value	Sarcopenia (*n* = 47)	Non-Sarcopenia (*n* = 85)	*P* value
TMTs (s)	200.96 ± 16.56	198.01 ± 21.54	0.38	212.32 ± 24.91	201.69 ± 27.78	**0.04**
DST (digits)	7.40 ± 1.13	7.25 ± 1.47	0.53	7.19 ± 1.99	7.08 ± 2.02	0.77
DSST(scores)	78.83 ± 8.41	79.12 ± 5.57	0.83	74.36 ± 10.85	78.76 ± 8.16	**0.02**
CDT (pts)	4.23 ± 0.81	4.26 ± 0.73	0.86	3.81 ± 1.27	4.25 ± 0.67	**0.03**

Significant values are in [bold]

*TMTs* the trail making tests, *DST* digit span test, *DSST* digit symbol substitution, *CDT* clock drawing test, *pts* points

### Relationship between sarcopenia and early POCD

As demonstrated in Table 4, our findings revealed that all the independent variables exhibited no significant multicollinearity (with all Tolerance values exceeding 0.1 and VIFs below 10).

**Table 4:** Collinearity analysis of related variables included in multivariate logistic regression analysis

Variables	Tolerance	Variance inflation factor
Age ≥ 80 years	2.496	1.040
BMI<18.5 kg/m2	0.928	1.072
Hs-CRP > 6 mg/L	2.016	1.161
Plasm Ceramide (22:0)	0.668	1.142
Sarcopenia, n (%)	2.675	1.072

*BMI* Body Mass Index, *Hs-CRP* High-sensitivity C-reactive protein, *ASA* American Society of Anesthesiologists

The preliminary results from the single-variable logistic regression analysis revealed that individuals aged 80 or above, an Hs-CRP > 6 mg/L, and the occurrence of sarcopenia were all linked to a higher risk of early POCD. Furthermore, the findings from the multivariate logistic regression analysis, presented in Table 5, revealed that preoperative sarcopenia (OR:3.716, 95%CI 1.492–9.254, *P* = 0.005) was significantly associated with early POCD, after controlling for other variables.

**Table 5:** Risk factors for postoperative cognitive decline according to univariate and multivariate logistic regression analyses

Variable	Univariate analyses		Multivariate analysis	
	OR(95%CI)	*P* value	OR(95%CI)	*P* value
Age ≥ 80 years	3.020(1.306–6.988)	**0.010**	3.364(1.343–8.427)	**0.010**
Sarcopenia	3.776 (1.618–8.814)	**0.002**	3.716(1.492–9.254)	**0.005**
BMI<18.5 kg/m2	2.283(0.808–6.449)	0.119		
Hs-CRP > 6 mg/L	3.600(1.546–8.385)	**0.003**	3.229(1.308–7.968)	**0.011**
Plasm ceramide (22:0)	1.118(0.998–1.253)	0.055		

Significant values are in [bold].

*BMI* Body Mass Index, *Hs-CRP* High-sensitivity C-reactive protein

**Fig. 3:**
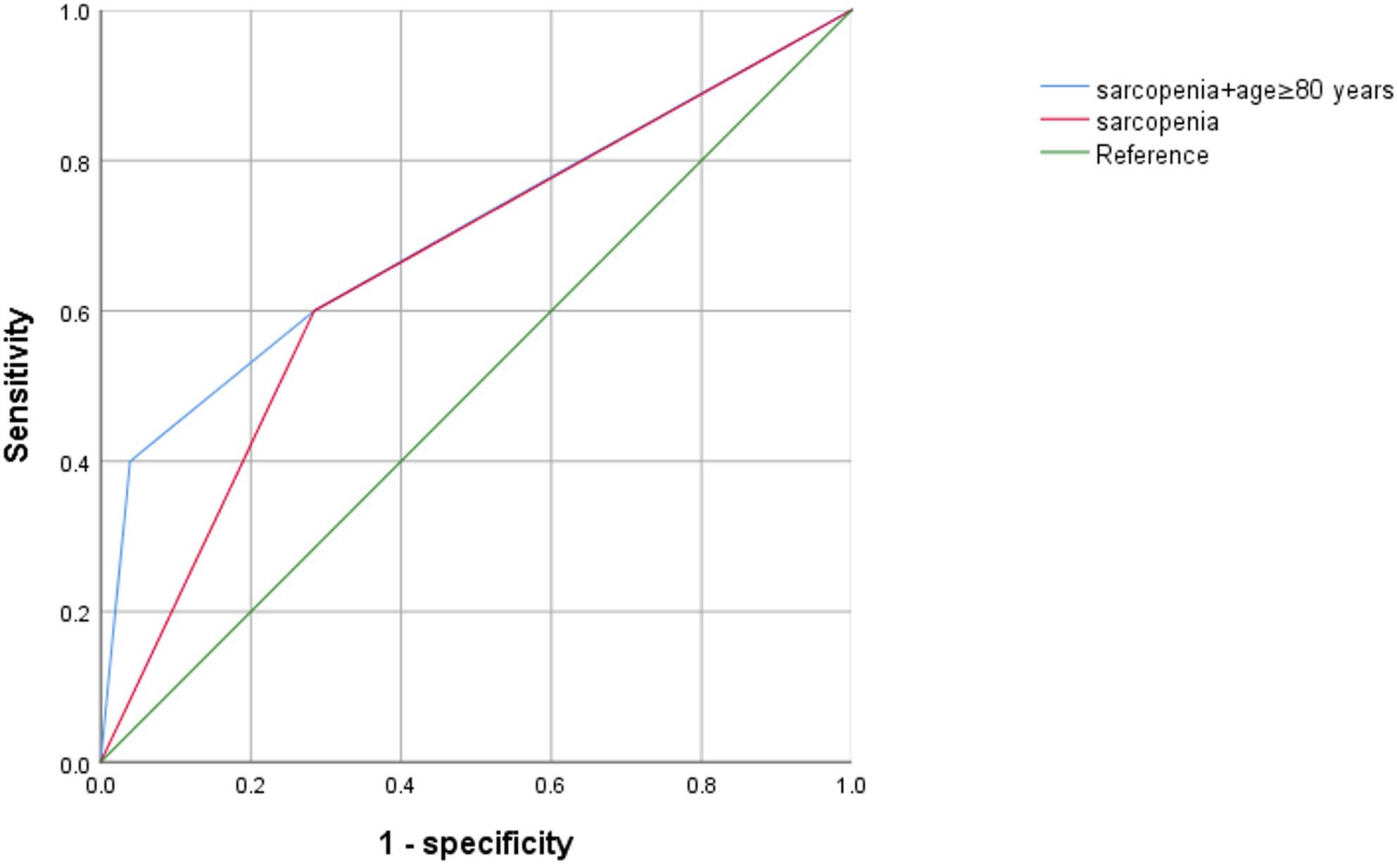
ROC analysis of the predictive value of preoperative sarcopenia and sarcopenia + Age ≥ 80 years for early POCD

As shown in Table 6; Fig. 3, The ROC curve’s area for predicting early POCD based on preoperative sarcopenia was 0.658 (95%CI 0.543–0.772, *P* = 0.009); based on sarcopenia combining with Age ≥ 80 years was 0.704 (95%CI 0.584–0.823, *P* = 0.001).

**Table 6:** The AUC of the subjects with different risk factors and combined risk factors

Variable	Statistical value AUC (95%CI) *P* value		YOUDEN	Cut-off	Sensitivity (%)	Specificity (%)
Age ≥ 80 years	0.629(0.512–0.747)	**0.032**	0.258	0.5	53.3	72.5
Sarcopenia	0.658(0.543–0.772)	**0.009**	0.316	0.5	60	71.6
Hs-CRP > 6 mg/L	0.653(0.538–0.767)	**0.011**	0.306	0.5	60	70.6
Sarcopenia + Age ≥ 80 years	0.704(0.584–0.823)	**0.001**	0.361	0.43	40	96.1
Sarcopenia + Hs-CRP > 6 mg/L	0.742(0.654–0.831)	**0.000**	0.492	0.17	93.3	55.9
Age ≥ 80years + Hs-CRP > 6 mg/L	0.716(0.612–0.819)	**0.000**	0.353	0.17	83.3	52

Significant values are in [bold]

*Hs-CRP* High-sensitivity C-reactive protein

### Discussion

The prospective design of our study in elderly patients undergoing hip fracture surgery enables a more direct examination of the association between sarcopenia and early POCD in this high-risk population. Sarcopenia was significantly associated with early POCD (OR: 3.716, 95% CI: 1.492–9.254, *P* = 0.005). This correlation was robust to adjustment for age, inflammation, or any other confounding variables.

Early POCD is a frequently encountered complication following hip fracture surgery in older adults. Analysis of clinical data revealed that age, preexisting cognitive disorders, and infection were consistently associated with an increased risk of postoperative cognitive dysfunction [39, 40]. As far as we know, this was the first observational cohort study to explore the relationship between sarcopenia defined by AWGS2019 criteria and CT-based T12 measurement and POCD after hip fracture surgery. Moreover, we sought to define the diagnostic criteria for early POCD using the outcomes of neuropsychological assessments relevant to clinical outcomes. This research found that preoperative sarcopenia experienced a higher incidence of early POCD (38.3% vs. 14.1%, *P* = 0.003), particularly in executive function and attention domains. These findings are consistent with those from a previous study [41]. Nonetheless, the incidence of cognitive decline after surgery was found to be less than what was noted in an earlier study [42], probably because the participants in our research were generally younger.

Earlier research has demonstrated a strong connection between sarcopenia and the deterioration of attention and executive function, but there is no significant correlation with short-term memory changes [43]. Our findings are consistent with this pattern. Specifically, patients with sarcopenia in our cohort showed significant decline on postoperative day 7 in test of executive functions, processing speed and complex attention (TMTs, DSST, and CDT; *P* < 0.05). In contrast, the DST test results did not show any differences. Sarcopenia decreased postoperative executive functions, processing speed and complex attention in elderly hip fracture patients manifested by the decline in test scores of TMTs, DSST and CDT; as these three tests all require patients to complete the tests through coordinated hand-writing operations. however, its impact on short-term memory function is not significant; furthermore, the DST results also do not show any obvious changes.

Sarcopenia is associated with an increased risk of early POCD. Although the underlying pathophysiological mechanisms of early POCD remain unclear and effective preventive strategies are not yet well defined, recognizing related risk factors could be a crucial initial step in preventing this condition among elderly patients. Earlier research revealed various potential factors for cognitive decline, such as older age, chronic pain before surgery, C-reactive protein levels before surgery, high ASA physical status, low BMI and postoperative serum CHI3L1 levels [41, 44–46]. The mechanism by which sarcopenia increases the risk of postoperative cognitive decline remains incompletely understood. Currently, it is considered to be the result of the synergistic action of multiple factors, primarily centered around systemic inflammatory responses and enhanced oxidative stress, dysfunction of sphingolipid metabolism, and abnormalities in the neuromuscular axis.

### Systemic inflammatory responses and enhanced oxidative stress

Surgical trauma can trigger the body’s stress response, leading to elevated levels of inflammatory factors (such as IL-6, TNF-α) and accumulation of oxidative stress products [47]. These pathological physiological changes are considered to be an important mechanism for POCD. Patients with sarcopenia often have a chronic low-grade inflammatory state, which is related to elevated serum CRP levels [48], and due to the reduction in muscle mass, their ability to clear inflammatory mediators and cope with oxidative stress also decreases. On this basis, preoperative chronic inflammation, in conjunction with acute inflammation resulting from surgical injury further intensifies, forming a synergistic amplification effect. Excessively released inflammatory factors can enter the central nervous system by damaging the integrity of the blood-brain barrier, causing neuronal damage, synaptic dysfunction, and imbalance of neurotransmitters, ultimately promoting the occurrence and development of cognitive dysfunction [49], while an increase in Hs-CRP concentration in the elderly population predicts a decline in executive power and memory [50]. DuPont [51] and colleagues discovered a strong association between elevated CRP levels and the development of sarcopenia, along with an inverse relationship between SMI and preoperative Hs-CRP levels. In individuals suffering from sarcopenia, adipose tissue infiltrates skeletal muscle leading to growth and increase of adipocytes. This process is accompanied by infiltration of proinflammatory macrophages and various immune cells which results in heightened levels of circulating proinflammatory substances [52]. Our results revealed that baseline Hs-CRP >6 mg/L (OR = 3.229, 95%CI 1.308–7.968, *P* = 0.011) indicates a high risk for early POCD.

### Dysfunction of sphingolipid metabolism

Sphingolipids, as an important component of cell membranes, are involved in regulating the proliferation, differentiation and apoptosis processes of muscle cells [14]. Abnormal metabolism of sphingolipids (such as abnormal accumulation of ceramides) can lead to impaired muscle cell function, inhibit the synthesis of muscle proteins, accelerate muscle loss, and directly promote the occurrence and development of sarcopenia [53]. Postoperative stress (such as inflammation, ischemia, etc.) can induce abnormal sphingolipid metabolism, leading to the accumulation of toxic sphingolipids such as ceramide in brain tissue. Such accumulation can disrupt the integrity of the blood-brain barrier, trigger neuroinflammatory responses, and damage neuronal synaptic function, ultimately resulting in cognitive impairment [54]. As indicated in prior research that has shown that sphingolipids, especially ceramide, may play a role in the decline of cognitive function [55]. Moreover, earlier research showed that there is an independent link between plasma ceramide and cognitive deterioration [56]. However, our findings suggest that plasma ceramide (22:0) was not an independent risk factor for early POCD (OR:1.118, 95%CI 0.998–1.253, *P* = 0.055). This might be attributable to the presence of additional potential confounding factors associated with ceramide (22:0), such as aging and sarcopenia [10, 57]. When these variables are accounted for in the multivariate model, they may attenuate the independent association between ceramide (22:0) and cognitive outcomes. Sarcopenia alters the relationship between increased plasma ceramide concentrations and early POCD. Future research will continue to explore intervention strategies aimed at modulating ceramide metabolism in patients with sarcopenia and elucidating the relationship between ceramide accumulation and early POCD.

### Abnormalities in the neuromuscular axis

Muscles are not only organs for movement but also an important endocrine organ, capable of secreting various muscle factors (such as irisin, brain-derived neurotrophic factor) [58]. These muscle factors play a crucial role in promoting the survival, proliferation, and synaptic plasticity of neurons, and they are of great significance for maintaining normal cognitive functions. In patients with sarcopenia, due to the decline in muscle mass, the synthesis and secretion of muscle factors decrease, resulting in a weakened protective effect on the central nervous system [13]. At the same time, the decline in muscle function leads to a reduction in physical activity, which further inhibits the release of muscle factors; thus forming a vicious cycle of “sarcopenia - reduced muscle factor secretion - impaired neuroprotective function”, significantly increasing the risk of postoperative cognitive dysfunction [58]. Our research group will designate the study of the neuromuscular axis and muscle factors as one of the key directions for our future work.

Our results revealed that sarcopenia (OR: 3.716, 95% CI 1.492–9.254, *P* = 0.005), age ≥ 80 years (OR: 3.346, 95% CI 1.343–8.427, *P* = 0.01) and preoperative Hs-CRP > 6 mg/L (OR: 3.229, 95% CI 1.308–7.968, *P* = 0.011) were significantly associated with an increased risk of early POCD after adjusting for potential confounding factors.

A number of studies have indicated that the presence of sarcopenia or frailty before surgery is linked to complications and clinical outcomes undergoing hip surgery in elderly [59, 60]. Frailty and sarcopenia are age-related conditions that exhibit a concomitant relationship, as they share many common clinical features and etiological factors. Frailty, a decline in physiologic reserve commonly found in older adults, refers to a state of increased vulnerability to stressors that develops as a multisystemic derangement independent of chronological aging or specific diseases and predisposes individual to numerous negative health-related events [61]. The pathophysiology of frailty and sarcopenia resembles that of other prototypical geriatric conditions, for which a multifactorial etiology is at play involving many biological hallmarks of aging (i.e., genomic and epigenetic instability, loss of proteostasis, mitochondrial dysfunction, telomere shortening, dysregulated nutrient signaling, stem cell exhaustion, cellular senescence, and altered intercellular signaling) [62]. In clinical studies there is a significant correlation between sarcopenia and cognitive decline in elderly patients after surgery [63, 64]. Although sarcopenia is one of the core components of frailty, frailty involves not only the muscle dysfunction manifested by sarcopenia but also often accompanies various systemic abnormalities such as cognitive decline, nutritional imbalance, decreased immune function, and chronic inflammatory state. Therefore, individuals with only muscle function impairment but without a decline in multiple physiological reserves may not conform to the overall phenotype of frailty. Sarcopenia can serve as an effective proxy indicator for frailty, but it still cannot completely replace a comprehensive assessment of frailty in clinical practice. To more accurately identify and manage frail patients, a comprehensive judgment should be made by combining multiple assessment tools and methods. Our findings suggest that having sarcopenia before surgery is significantly associated with cognitive decline and shows a moderate discriminatory ability in identifying patients with early POCD (AUC = 0.658, 95% CI 0.543–0.772, *P* = 0.009). This finding not only supports but also expands upon previous studies that explored the connection between low skeletal muscle mass and early POCD. Furthermore, when age ≥ 80 years is considered alongside sarcopenia, the discriminatory ability improves, as indicated by an AUC_ROC_ of 0.704(95% CI: 0.584–0.823, *P* = 0.001), which aids in better identifying patients who may be at high risk for early POCD.

Sarcopenia was identified using the guidelines set forth by AWGS 2019. CT has been recognized as a valid tool for diagnosing sarcopenia in recent studies [65]. Sarcopenia diagnosed based on CT at the L3 level is strongly correlated with diagnoses made via BIA and DXA [20, 66]. Moreover, sarcopenia diagnosis using CT at the T12 level is highly correlated with both L3 level [CT] assessment and BIA [67, 68]. Collectively, these findings provide considerable evidence supporting the feasibility and representativeness of diagnosing sarcopenia via chest CT scans at the 12th thoracic vertebra. During the study period, CT scans were routinely utilized in clinical practice for evaluating pulmonary conditions, including COVID-19 screening, without additional costs. However, given that patients with hip fractures are unable to ambulate prior to surgery, we did not conduct the 3-meter timed test for physical performance.

Sarcopenia and early POCD are age-related disorders that share risk factors such as hip fracture [69]. In this report, the prevalence of sarcopenia was approximately 35.6% as per the AWGS 2019 definition with CT-based T12 measurement, which is comparable to that reported in a previous study where the prevalence was 37% based on the criteria recommended by the European Working Group on Sarcopenia in Older People (EWGSOP) [70]. Nonetheless, several studies have reported a lower occurrence of sarcopenia, with figures of 22.6% [59] and 24.3% [71], when compared to our findings. These variations may be ascribed to dissimilarities in diagnostic criteria, the populations studied, and the sample sizes utilized.

Our study has several limitations. Although our study employs rigorous inclusion and exclusion criteria to guarantee data quality, our sample size remains relatively limited, and we only examined the factors linked to early cognitive decline within 7 days post-surgery, which may not capture the dynamic changes in cognition during the entire postoperative period. In future studies, we will be able to continuously monitor the alterations in patients’ cognitive function over an extended period, which can range from one month, three months, to one year. Secondly, since the population of our study was confined to patients undergoing hip fracture surgery, our findings might not be applicable to patients undergoing other types of surgeries. Third, the small sample size restricted the variety of factors that could be incorporated into the multiple analyses. Several additional risk factors for cognitive decline after surgery, including frailty, chronic pain prior to the operation, sleep disturbances before surgery, and hypothermia during the perioperative period were not included in our analysis. Finally, A limitation of our study is the lack of blinding of the cognitive assessors to the patients. However, the use of objective neuropsychological tests with standardized protocols and the extensive training provided to all assessors are likely to have minimized the potential for observer bias, supporting the robustness of our primary findings.

## Conclusion

Our findings suggest that sarcopenia before surgery is significantly associated with cognitive decline following the procedure. In conjunction with age ≥ 80 years, it enhances a useful clinical profile for risk stratification. This study highlights the importance of considering sarcopenia in preoperative assessments, which could facilitate targeting early interventions to patients who are most likely to benefit and may help in reducing the incidence of early POCD. For subsequent studies, it is essential to carry out long-term and broad prospective cohort studies that include diverse age groups and types of surgery in order to verify the effect of sarcopenia on early POCD.

## Data Availability

The data that support the findings of this study are included in the article; further inquiries can be directed to the corresponding author. E-mail: [huxianwen001@163.com]

## Abbreviations

ASA: American Society of Anesthesiologists
AUC_ROC_: the area under the receiver operating characteristic curve
AWGS: Asia Working Group of Sarcopenia
BMI: Body mass index
CDT: Clock drawing test
CIs: confidence intervals
COPD: Chronic obstructive pulmonary disease
DST: Digit span test
DSST: Digit symbol substitution test
EPV: events per variable
EWGSOP: European Working Group on Sarcopenia in Older People
HGS: hand grip strength
Hs-CRP: High-sensitivity C-reactive protein
HU: Hounsfield Unit
MMSE: Mini-Mental State Examination
ORs: odds ratios
POCD: Postoperative cognitive decline
ROC: receiver operating characteristic
SAS: Self-rating Anxiety Scale
SDS: Self-rating Depression
SMA: Skeletal muscle area
SMI: Skeletal muscle index
TMTs: Trail making tests
VIF: variance inflation factor
